# Challenges in Neuroimaging in COVID-19 Pandemia

**DOI:** 10.3389/fneur.2020.579079

**Published:** 2020-11-30

**Authors:** Sofía González-Ortiz, Santiago Medrano, José María Maiques, Jaume Capellades

**Affiliations:** Neuroradiology Section, Radiology Department, Hospital del Mar, Barcelona, Spain

**Keywords:** neuroradiology, radiology, tele-radiology, personal protective equipment, COVID-19

## Introduction

Since the first case of infection was reported in December 2019, in Wuhan, China, SARS CoV 2 has spread all over the world, and was declared as a pandemia on the 11th of March by the WHO. The reported mortality rate is between 0.3 and 1% in the general population, rising to 14% in hospitalized cases (1). Even though Covid-19 infection causes a predominantly respiratory disease, its explosive eruption worldwide has affected all medical specialties.

Health care systems and workers have had to react rapidly. Each region and hospital has adapted differently depending on their specific characteristics, the prevalence of the infection and the recommendations of governments and preventative medical services. The practice of Neuroradiology, along with Radiology departments, have not escaped the effects and have had to face up to the new circumstances (2). Some works (articles, webinars and guidelines) have appeared giving recommendations and sharing their experience to face the challenge that the Covid-19 pandemia implies for the Neuroradiology. In this article, we present and discuss these recommendations in the different phases of the pandemia.

## Before The Peak of The Pandemia

In the early stages of the pandemia, crisis committees, connected with the local, regional and state public institutions, were created to establish new guidelines and protocols for each center (3–7). A general practice adopted in Radiology and Neuroradiology, was the creation of departmental co-ordination groups (typically comprising a radiologist/neuroradiologist, a radiographer and a secretary), to work in conjunction with these committees (8–10). In addition, general measures were implemented to limit the exposure of healthcare workers and patients and for early viral detection. Securing the supply of medical material and personal protective equipment (PPE) was also a priority (6).

As the rapid and explosive spread of the Covid-19 infection required a rapid response, this coordination and reorganization of Radiology departments, a common strategy followed in hospitals, was, in our opinion, key to achieving this. The supply of PPE for staff, another critical point during the early stages of the pandemia, was a great challenge, due to the high worldwide demand (11).

## The Peak of The Pandemia

In this phase various measures have been recommended.

One of these is the strict selection of neuroimaging tests. Although each center has had to set their own criteria depending on their particular idiosyncrasies, there have been some general recommendations (4, 5, 12, 13). In the case of critical examinations, where the neuroimaging could impact the immediate management of patients, the recommendation has been to perform the test despite the pandemic situation, subject to a risk/benefit analysis. In the case of non-critical neuroradiological examinations, the recommendation has been to postpone them and establish levels of priority (13–16). In some cases, examinations could even be canceled (15).

In this phase, the increased pressure on hospitals due to the number of Covid patients, with the consequent lack of material and human resources, and the need for social distancing, has made it impossible to carry out the usual volume of examinations. For this reason, even if there have been no specific recommendations on which particular neuroradiological examinations to maintain, we believe that the prioritization of tests during the peak of the pandemia has been key to ensure that the most critical patients received an optimal radiological diagnosis. The establishment of different priority levels in the elective tests has been essential for their orderly rescheduling. To give an objective view of the impact, neuroradiological examinations during the pandemic peak decreased by almost 50% (17, 18). We think it has also been important, as emphasized in some articles, the need of a fluid communication between neurologists, neurosurgeons and other clinicians, to highlight any special situations arising in particular cases.

Special mention should be made of patients with acute stroke, who present a particular challenge for neuroradiology departments, due to the existing relationship reported between patients with severe coronavirus infection and cerebrovascular stroke disease (19). As these patients usually undergo a brain CT and angio-CT scan, some studies have recommended the incorporation of a chest CT to rule out the possible existence of a concomitant pneumonia due to Covid-19, which would require isolation of the patient (20, 21). It seems a sensible recommendation when the prevalence of the infection in the population is high.

In terms of patient protection, the first step has been to detect potential cases in patients coming for a neuroradiological test. To this end screening questionnaires (3–5, 9) have been carried out, often even conducted by telephone before the arrival of the patient, followed by PCR tests if necessary and available. Specific circuits have been established within Neuroradiology departments to avoid contact between infected and uninfected patients. “Clean” radiological equipment has been kept for uninfected patients and “dirty” for infected patients (5, 13, 22–25). Social distancing has been enforced in waiting rooms and masks made mandatory for all patients (5, 13, 26). Cleaning, disinfection and air purification frequency have also been increased (5, 13, 22–25).

These are reasonable measures which are recommended in guidelines and have been adopted generally in hospitals and imaging centers. We think it is important that each hospital establishes their own protocols, as these recommendations can be carried out in different ways according to particular characteristics. For example, in relation to air purification, some of the recommended measures have been the use of a high-efficiency particulate air (HEPA) filter, ultraviolet irradiation or simply lengthening the time between two patients. The choice as to which to use is a decision that depends on multiple factors. In relation to the use of masks or other medical devices, such as ventilators, in Neuroradiology departments, we think it is important to highlight that they need to be compatible with the MRI environment, for both safety and image quality reasons. In the case of CT examinations, they must not contain metallic elements which could distort the image (26–29).

In terms of healthcare staff protection, education about security measures, the provision of PPE and the establishment of physical barriers, such as plastic screens and equipment covers, have been some of the more extensively adopted precautionary measures (5, 13, 14). Tele-neuroradiology has been another widely adopted practice to reduce the exposure of departmental staff, with the use of “Picture Archiving and Communication System” PACS. Where telematic work has not been possible, the establishment of groups, working different hours or days, has been an extensively used option, along with the use of individual workstations and maintaining social distancing in the work-space (7, 26, 29). In order to maintain clinical and educational communication, the use of phone calls (instead of personal interactions) and teleconference applications for virtual sessions has been widespread, especially for essential clinical care meetings (30). These applications allow communication from workstations or even phones, and also screen sharing to show neuro-radiological images (25).

Probably one of the most specific challenge for Neuroradiology, related to the staff protection, has been the rapid deployment of Home PACS Workstations and the expansion of teleradiology (31–33).

## After The Peak of The Pandemia

Once the peak of the pandemic has passed, the most emphasized recommendation for Radiology departments has been to recover activity in a tiered manner (13–15, 34–36). The postponed examinations should be re-scheduled following the degrees of priority established during the peak of the pandemia (13–15). The new petitions generated by the recovery of activity in hospitals also need to be taken into account. We think that all these common measures to recover radiological activity, have to be adapted to each situation, as the prevalence of the pandemia and the resources of health care systems could be very variable. In this regard we found the work of Madhuripan et a. (17) interesting, which related the radiology volume recovery after the pandemia to different variants, such as regional pandemic severity, the lifting of government restrictions, patient Covid-19 infection concern, management during the pandemic peak, impact of the economic recession and Radiology practice profile.

General measures to avoid the transmission of Covid-19 have still been recommended in this phase and are likely to be necessary for some time (35). For example, the obligatory use of masks and enforcing of social distancing in the hospital, the use of PPE for health workers and the increased disinfection and ventilation of imaging suites. As a result of these measures, Radiology departments still need to allow for longer times between patient examinations. Many hospitals have responded to this by increasing the hours of radiological assistance, extending the activity of the MRI and CT scans during the night and weekend shifts (34, 35). We think this may be necessary to re-schedule all the postponed activity, but hospital management must take into account that it may mean hiring more staff or agreeing new shifts with workers.

The continued use of tele-radiology, at least partially, is still recommended at this stage (13, 34, 35). This has been one of the most widespread measures adopted in neuroradiology and has generally been implemented successfully (31–33). After these experiences and in line with other articles (37), we believe that for neuroradiologists, the coronavirus pandemic may contribute to the permanent establishment of tele-neuroradiology, or at least to a mixed model with part of the time physically present and part of the time reporting remotely.

## Conclusion

The particular challenges for the practice of Neuroradiology during the Covid pandemia have been different during the distinct phases. In the early stage, the main challenge was the need for a rapid response. During the peak of the pandemia, the challenge was to maintain critical neuroimaging assistance, whilst preventing the spread of the infection amongst patients and healthcare workers. After the peak of the pandemia the challenge has been to recover neuroradiological activity while maintaining some Covid-19 measures, which seem likely to continue for a while. Some of the strategies with which Neuroradiology has faced the challenges of each phase have been general, and others more specific to the specialty. Broadly they have been quite consistent throughout the different articles and guidelines published.

After the peak of the Covid-19 pandemia we have to stay alert and know how to react on time to possible next waves, using what we have already learnt during these months. Neuroradiology assistance should be maintained taking into account the general care of the patients and the global health situation.

## Author Contributions

All authors contributed to the article and approved the submitted version.

## Conflict of Interest

The authors declare that the research was conducted in the absence of any commercial or financial relationships that could be construed as a potential conflict of interest.
